# Mr. Lynam, on the Aqua Ammoniæ Acetata

**Published:** 1799-10

**Authors:** Charles Lynam

**Affiliations:** No. 48, Aldermanbury


### *To the Editors of the Medical and Phyſical Journal.*

Gentlemen,

As there are many practitioners in the daily habit of uſing the *aqua ammoniæ acetata* of the Diſpenſary, and who are much attached to its uſe as a febrifuge medicine, the powers of which are conſiderably increaſed, and its efficacy improved, by the following mode of preparing it, I truſt it will be found worthy of being inſerted in your very uſeful and valuable Journal. I think it a duty incumbent upon me, to ſubmit it to the medical practitioners, aſſuring them that it has all the efficacy of the former medicine, in addition to new acquired properties, one of which is its being infinitely more pleaſant and agreeable to the taſte.

I am, Gentlemen, with great reſpect,

